# The Lysosomal Membrane Protein Lamp2 Alleviates Lysosomal Cell Death by Promoting Autophagic Flux in Ischemic Cardiomyocytes

**DOI:** 10.3389/fcell.2020.00031

**Published:** 2020-02-07

**Authors:** Lin Cui, Li-Ping Zhao, Jing-Ying Ye, Lei Yang, Yao Huang, Xu-Pin Jiang, Qiong Zhang, Jie-Zhi Jia, Dong-Xia Zhang, Yuesheng Huang

**Affiliations:** ^1^Institute of Burn Research, State Key Laboratory of Trauma, Burns and Combined Injury, Southwest Hospital, Army Medical University (Third Military Medical University), Chongqing, China; ^2^Friendship Plastic Surgery Hospital, Nanjing Medical University, Nanjing, China

**Keywords:** Lamp2, LMP, autophagic flux, cardiomyocytes, OGD

## Abstract

Lysosomal membrane permeabilization (LMP) has recently been recognized as an important cell death pathway in various cell types. However, studies regarding the correlation between LMP and cardiomyocyte death are scarce. Lysosomal membrane-associated protein 2 (Lamp2) is an important component of lysosomal membranes and is involved in both autophagy and LMP. In the present study, we found that the protein content of Lamp2 gradually decreased in response to oxygen, glucose and serum deprivation (OGD) treatment *in vitro*. To further elucidate its role in ischemic cardiomyocytes, particularly with respect to autophagy and LMP, we infected cardiomyocytes with adenovirus carrying full-length Lamp2 to restore its protein level in cells. We found that OGD treatment resulted in the occurrence of LMP and a decline in the viability of cardiomyocytes, which were remarkably reversed by Lamp2 restoration. Exogenous expression of Lamp2 also significantly alleviated the autophagic flux blockade induced by OGD treatment by promoting the trafficking of cathepsin B (Cat B) and cathepsin D (Cat D). Through drug intervention and gene regulation to alleviate and exacerbate autophagic flux blockade respectively, we found that impaired autophagic flux in response to ischemic injury contributed to the occurrence of LMP in cardiomyocytes. In conclusion, our present data suggest that Lamp2 overexpression can improve autophagic flux blockade probably by promoting the trafficking of cathepsins and consequently conferring cardiomyocyte resistance against lysosomal cell death (LCD) that is induced by ischemic injury. These results may indicate a new therapeutic target for ischemic heart damage.

## Introduction

Cardiac ischemia, characterized by inadequate supply of oxygen and nutrients, often leads to irreversible damage to the myocardium, which is manifested as contractile tissue loss and compensatory cardiac hypertrophy. Many pathological conditions, such as coronary heart disease, and heart diseases caused by other factors, including long residence in plateau regions, severe burns, traumatic hemorrhage and organ transplantation, lead to ischemic injury (Edtinger et al., 2014; Duke et al., 2016). Therefore, a better understanding of the mechanisms underlying cardiac loss could yield new therapeutic targets against these pathological conditions.

Lysosomes were initially called “suicide bags” by Christian de Duve more than 50 years ago owing to the many powerful hydrolases they contain (de Duve, 2005). Lysosomes have been regarded as the central coordinator of cellular clearance and energy metabolism due to their vital control over autophagy and metabolism signal molecules, AMPK and mTORC1 (Settembre et al., 2013; Milkereit et al., 2015; Lin and Hardie, 2018). In addition, lysosomes also regulate cell death signals. The most common cell death pathway associated with lysosomes is called lysosomal cell death (LCD) (Gomez-Sintes et al., 2016; Gaidt et al., 2017; Mai et al., 2017). LCD is characterized by lysosomal membrane permeabilization (LMP) and mediated by the release of lysosomal cathepsins into the cytoplasm, which causes caspase-dependent and caspase-independent cell death (Windelborn and Lipton, 2008; Jiang et al., 2016; Clerc et al., 2018). To date, many pathogenic factors that induce LMP have been discovered, including ROS, lysosomotropic drugs, bacterial and viral products (de Duve, 2005; Huang et al., 2017; Malet et al., 2017). Studies regarding LMP in neuronal ischemic injury have revealed that ROS generation, calcium overload and calpain activation account for the induction of LMP and consequent neuronal death (Yamashima and Oikawa, 2009; Lipton, 2013). However, the correlation between LMP and myocardial ischemic injury is not as well established (Tiwari et al., 2008).

Macroautophagy, hereafter referred to as autophagy, is a highly regulated process involved in the degradation of protein aggregates and damaged organelles via the lysosomal system (Dikic and Elazar, 2018; Mizushima, 2018). The initiation of autophagy is indicated by the development of a double-layered, crescent-shaped membrane known as a phagophore, which elongates and matures into an autophagosome. The autophagosome sequesters and engulfs long-lived proteins and damaged organelles, which are subsequently degraded in lysosomes. The entire process is referred to as autophagic flux. Blocked autophagic flux is characterized by undigested macromolecules and accumulated autolysosomes, which can result in enlarged lysosomes and aggravate lysosome injury (Gonzalez et al., 2012). In turn, the induction of LMP is accompanied by the release of lysosomal protons and cathepsins into the cytoplasm, which can also cause the dysfunction of autophagic flux (Gabande-Rodriguez et al., 2014; Giuliano et al., 2015).

Lamp2 is a heavily glycosylated type-1 lysosomal membrane protein and an important regulator of autophagy. There are three different isoforms in humans and mice and only one isoform in rats. The human Lamp2 mutation causes Danon disease (Endo et al., 2015). Humans with Danon disease and Lamp2-knockout mice both show hypertrophic cardiomyopathy characterized by accumulated autophagic vacuoles filled with polymorphic contents, which are mainly the result of disrupted macroautophagy (Tanaka et al., 2000; Rowland et al., 2016). In addition, studies have revealed that Lamp2 plays a role in cell survival. Upregulation of Lamp2 in the plasma membrane is induced by chronic acidosis to protect cancer cells from acid-induced hydrolysis (Damaghi et al., 2015) and to promote their survival via chaperone-mediated autophagy (Saha, 2012). However, loss of Lamp2 aggravates oxidative stress through the obstruction of ROS clearance (Law et al., 2016; Qin et al., 2017).

Here, to learn more about the role of Lamp2 in the ischemic heart, we studied whether it can act as a stress protectant and determined its underlying mechanisms. We found that ischemia/hypoxia robustly reduces the protein content of Lamp2 and that restoration of this protein significantly enhances the trafficking of lysosomal cathepsins and reverses the autophagic flux blockade induced by ischemia/hypoxia treatment, promoting the resistance to LCD and protecting cardiomyocytes against ischemic injury.

## Materials and Methods

### Cardiomyocytes Culture and Oxygen, Glucose and Serum Deprivation

All animal experiments were approved by the Animal Experiment Ethics Committee of the Third Military Medical University and performed according to the Guide for the Care and Use of Laboratory Animals published by the US National Institutes of Health (NIH Publication, 8th Edition, 2011). Neonatal Sprague-Dawley rats (1–3 days old) were obtained from the Animal Center of the Third Military Medical University. Neonatal rat ventricular cardiomyocyte culture was performed according to the protocols published previously (Hu et al., 2010). For the OGD treatment, serum- and glucose-free medium was replaced before subjecting the cells to hypoxic conditions in a CO_2_ incubator (3131, Thermo Scientific) filled with 94% N_2_, 5% CO_2_ and 1% O_2_ for the indicated periods. The control cells were incubated in medium containing serum and glucose in a humidified atmosphere with 5% CO2 at 37°C for the same periods.

### Adenovirus Infection

Adenoviruses carrying full-length Lamp2 were purchased from OBiO Technology (Shanghai, China), and MCMV-null adenoviruses were used as negative controls. Infection efficiency was determined by Western blotting, and all experiments were performed 24–72 h after the cells were transfected. mCherry-GFP-LC3 adenoviruses were purchased from Hanbio Biotechnology (Shanghai, China). After infection for 24–72 h, the cardiomyocytes were subjected to experimental treatments, and images were captured with a confocal microscope (TCS-NT, Leica, Wetzlar, Germany).

### Gene Silencing With siRNAs

Lamp2 siRNAs, Cat D siRNAs and ATG5 siRNAs were all purchased from GenePharma (Shanghai, China). Cardiomyocytes were transfected with the targeting siRNAs or the negative control siRNAs with Lipofectamine 2000 (Invitrogen, United States) referring to the manufacture’s instructions. All experiments were performed after transfection for 36–72 h.

### Western Blotting Assay

Cardiomyocytes were harvested in RIPA buffer with protease inhibitor tablets and sonicated on ice. The lysate was then centrifuged at 14,000 rpm at 4°C for 15 min, and the supernatant was reserved. Protein concentrations were determined using Quick Start^TM^ Bradford 1x dye reagent (#500-0205, Bio-Rad, United States). Proteins were separated on an SDS-PAGE gel (Bio-Rad) and transferred to PVDF membranes (Millipore, United States), where they were blocked with 5% skim milk. Then, the membranes were incubated at 4°C overnight with the corresponding primary antibodies and HRP-conjugated secondary antibodies. Specific protein bands were visualized using Western Bright Sirius chemiluminescent HRP substrate (Pierce, United States) with a ChemiDoc XRS image detector (Bio-Rad). The following antibodies were used in this experiment: rabbit polyclonal anti-LC3B (L7543, Sigma-Aldrich, United States), anti-SQSTM1/p62 (5114, Cell Signaling Technology, United States), anti-Lamp2 (PA1-655, Invitrogen, United States), anti-Lamp1 (ab24170, Abcam), anti-ATG5 (12994, Cell Signaling Technology, United States), rabbit monoclonal anti-Cl-M6PR (ab124767, Abcam), and anti-CD-M6PR (ab134153, Abcam) and mouse monoclonal anti-beta-actin (ab8226, Abcam), anti-cathepsin D (sc-377124, Sana Cruz Biotechnology).

### Immunofluorescence and Confocal Microscopy

Cardiomyocytes were plated on glass coverslips, fixed with 4% paraformaldehyde for 10 min and blocked with 5% bovine serum albumin in PBS for 1 h at room temperature. Then, the cells were incubated with specific primary antibodies at 4°C overnight and were subsequently incubated with the corresponding secondary antibodies for 1 h at 37°C. The nuclei were stained for 5 min with DAPI. Cells were imaged using a confocal microscope. The following primary antibodies were used in this experiment: mouse monoclonal anti-galectin-3 (sc-25279, Santa Cruz Biotechnology) and goat polyclonal anti-DDDDK tag (ab1257, Abcam). The following secondary antibodies were purchased from Invitrogen: Alexa Flour-488 donkey anti-rabbit (A21206), Alexa Flour-568 donkey anti-mouse (A10037), and Alexa Flour-680 donkey anti-goat (A21084).

### Cell Viability and Toxicity Assays

Cell viability was determined with a Cell Counting Kit-8 (CCK- 8, C0037, Beyotime), and cytotoxicity was detected with a CytoTox-ONE^TM^ C homogeneous membrane integrity assay kit (G7890, Promega, United States), which is a fluorometric method used to measure the amount of lactate dehydrogenase (LDH) released into the medium from the non-viable cells. Both experiments were carried out following the manufacturer’s instructions. The value of OD450 normalized to that of the control group and the percentage of LDH released into the medium were used to reflect cell viability and the extent of the induced cytotoxicity, respectively.

### Acridine Orange Release Experiment

The change in the distribution of acridine orange was used to assess LMP, as previously reported (Lovejoy et al., 2011). Briefly, the cells were incubated with 10 μg/ml acridine orange for 15 min at 37°C, washed with prewarmed PBS three times before the medium was refreshed with new medium and then subjected to OGD treatment. The fluorescence images were captured with a confocal microscope.

### Cytosolic Cathepsin B Activity Measurement

To quantify the extent of LMP, we measured cytosolic Cat B activity as previously reported (Jaattela and Nylandsted, 2015; Repnik et al., 2016). First, we used a series of digitonin concentrations (0, 5, 10, 15, 20, 25, 30, 35, 40, 45, 50, and 200 μg/ml) to determine an appropriate concentration to extract the cytosolic fraction without lysosomes. The concentration of 200 μg/ml was used for the complete permeabilization of the cell membranes. Briefly, cardiomyocytes were plated on 24-well plates. After the desired treatment, the medium was removed, and each well was rinsed with PBS twice. Then, 200 μl of the digitonin dilution was added. The cells were then incubated on ice for 15 min on a rocking table (frequency 50/min) to allow for the reaction. The supernatant was reserved, and 50 μl of culture from each well was mixed with 50 μl of 30 μM cathepsin-specific fluorogenic substrate Z-RR-AMC (219392, Millipore) containing 5 mM DTT (Sigma-Aldrich) and seeded on a black 96-well plate with a transparent bottom. The kinetics of cathepsin activity were measured 30 min at 37°C with a Varioskan Flash microplate reader (Thermo Scientific) at an excitation wavelength of 380 nm and an emission wavelength of 460 nm. An LDH assay was performed on each well following the instructions (C0016, Beyotime). The appropriate concentration of the digitonin dilution was determined to be the one that generated the best possible permeabilization of the plasma membrane (LDH release) with minimal cathepsin release from the lysosomes. The extent of LMP in each group was determined by the percentage of cytosolic Cat B activity compared to the total activity at the appropriate concentration of digitonin (17 μg/ml) used to extract the cytosolic fraction.

### Autolysosome Detection

DALGreen fluorescent dye (D675, Dojindo Laboratories, Japan) was applied to detect autolysosomes in an experiment carried out according to the manufacturer’s instructions. Briefly, cardiomyocytes were incubated with a 0.5 μM DALGreen working solution at 37°C for 30 min before the supernatant was discarded. Then, the culture medium was washed twice. Images of the cells after experimental treatments were captured under a confocal microscope.

### Statistical Analysis

The statistical analysis was performed using SPSS software. The significance of differences between groups was evaluated by the unpaired Student’s *t*-test or one-way analysis of variance (ANOVA) followed by *post hoc* tests. A *P*-value < 0.05 was considered statistically significant for all comparisons.

## Results

### Lamp2 Protects Cardiomyocytes From Ischemic/Hypoxic Injury

**FIGURE 1:**
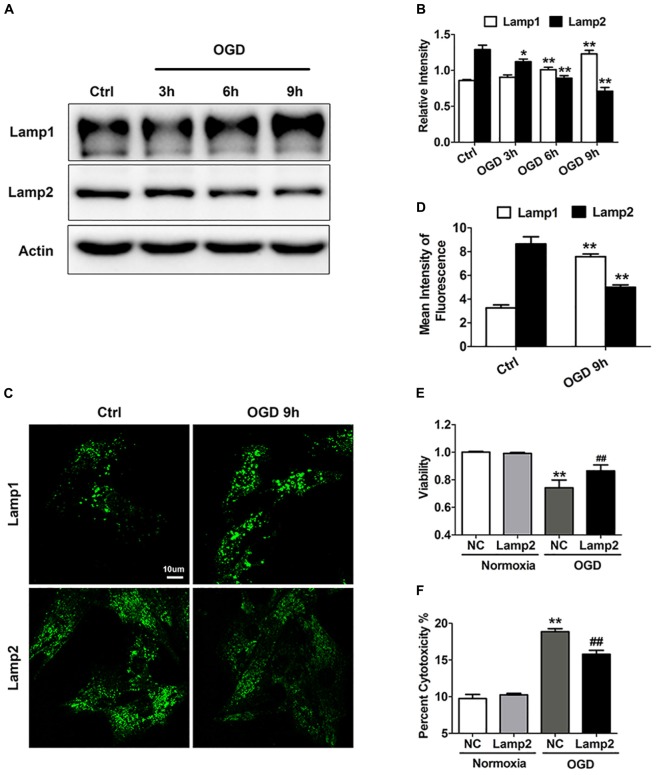
Lamp2 overexpression promotes resistance against OGD stress in cardiomyocytes. **(A)** Western blotting was performed to detect Lamp1 and Lamp2 levels after OGD treatment for different periods. **(B)** Quantitative analysis of the immunoblots in **(A)**. The data represent the mean ± SEM (*n* = 5). *P < 0.05 and **P < 0.01 versus the control group. **(C)** Representative confocal images of Lamp1 and Lamp2 after OGD treatment for 9 h. Scale bar, 10 μm. **(D)** Quantitative analysis of the fluorescence in **(C)**. Mean ± SEM. *n* = 3. **P < 0.01 versus the control group. **(E)** Cell viability was determined with a CCK-8 assay and was normalized to that of the control group. Mean ± SEM. *n* = 3. **P < 0.01 versus the normoxia + NC group, ^##^*P* < 0.01 versus the OGD + NC group. **(F)** LDH leakage analysis was performed to determine cell death. Mean ± SEM. *n* = 3. **P < 0.01 versus the normoxia + NC group, ^##^*P* < 0.01 versus the OGD + NC group.

Previous studies have indicated that lysosomal-associated membrane proteins (LAMPs) participate in various types of oxidative stress (Law et al., 2016; Qin et al., 2017). To investigate the role of LAMPs in ischemia-/hypoxia-induced cardiac injury, we used an *in vitro* OGD model to mimic the ischemic/hypoxic injury of cardiomyocytes and unexpectedly found opposite changes in these proteins. We observed a time-dependent increase in lysosomal-associated membrane protein 1 (Lamp1) but a time-dependent decrease in Lamp2 with the OGD treatment (Figures 1A,B). As shown in Figures 1C,D, the intensity of Lamp2 immunofluorescence significantly weakened. By contrast, the number of Lamp1 puncta increased and these puncta were brighter in the cells exposed to OGD stress, which might indicate an adaptation to activated autophagy. To explore whether the decrease in Lamp2 was involved in cardiac cell loss in response to OGD treatment, we used adenovirus carrying full-length Lamp2 to restore its protein content and Lamp2 siRNA to further downregulate its protein content (Supplementary Figure S1A). As shown in Figure 1E, exogenous expression of Lamp2 greatly reversed the reduction in cardiomyocyte viability induced by OGD treatment. In accordance, the leakage of LDH remarkably decreased with Lamp2 overexpression (Figure 1F). However, Lamp2 knockdown using siRNA had no significant effects on cell viability and cytotoxicity with OGD treatment but partially decreased cell viability in normal conditions (Supplementary Figures S1G,H). These data indicates that Lamp2 overexpression conferred cardiomyocyte resistance against ischemic/hypoxic injury.

### Lamp2 Alleviates LCD With OGD Treatment

**FIGURE 2:**
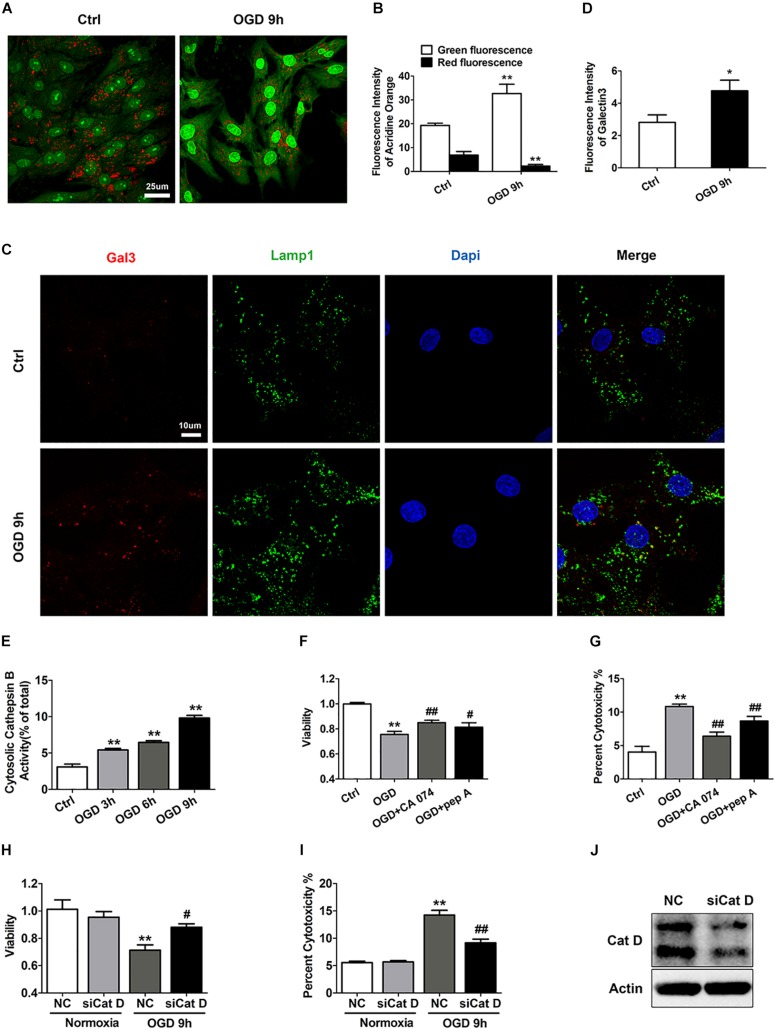
LMP is involved in ischemic/hypoxic injury of cardiomyocytes. **(A)** Representative images of the acridine orange staining after OGD treatment for 9 h. Bar, 25 μm. **(B)** Quantitative analysis of the images in **(A)**. Mean ± SEM. *n* = 5. **P < 0.01 versus the control group. **(C)** Gal3 immunofluorescence was used to determine lysosomal membrane injury. Bar, 10 μm. **(D)** Quantitative analysis of Gal3 immunofluorescence intensity. Mean ± SEM. *n* = 3. *P < 0.05 versus the control group. **(E)** Results from the extraction of the cytoplasm without lysosomes after OGD treatment for 3, 6, and 9 h with digitonin solution (17 μg/ml) to detect cathepsin B activity. Mean ± SEM. *n* = 3. **P < 0.01 versus the control group. **(F)** Cell viability was assessed by CCK-8 analysis, and the value was normalized to that of the control group. CA074 (20 μM) and pep A (12.5 μg/ml) alleviated the cell injury induced by OGD stress. Mean ± SEM. *n* = 3. **P < 0.01 versus the control group and ^#^*P* < 0.05, ^##^*P* < 0.01 versus the OGD group. **(G)** Cell injury was determined by LDH leakage analysis. Mean ± SEM. *n* = 3. **P < 0.01 versus the control group, ^##^*P* < 0.01 versus the OGD group. **(H)** Cat D siRNA improved cell viability as determined by CCK-8 analysis. Mean ± SEM. *n* = 3. **P < 0.01 versus the normoxia + NC group and ^#^*P* < 0.05 versus the OGD + NC group. **(I)** LDH leakage analysis was used to assess the cell injury. Mean ± SEM. *n* = 3. **P < 0.01 versus the normoxia + NC group, ^##^*P* < 0.01 versus the OGD + NC group. **(J)** Western blotting of Cat D was performed to detect the transfection efficiency of Cat D siRNA.

Given that Lamp2 is an abundant and important lysosomal protein, we aimed to further investigate whether the cardiomyocyte injury alleviated by Lamp2 restoration was correlated with lysosomal adaptation. We first aimed to clarify whether lysosomes were involved in the ischemic injury of the cardiomyocytes. The lysosomotropic dye acridine orange was applied to detect the integrity of the lysosomal membranes. As shown in Figures 2A,B, compared with the control group, the OGD group showed brighter green fluorescence and weaker red fluorescence, indicative of the release of acridine orange into the cytoplasm. To further corroborate that LMP occurs during OGD, we performed immunostaining for the marker of damaged endomembranes, galectin-3 (Gal3) (Maejima et al., 2013; Skowyra and Schlesinger, 2018). As shown in Figures 2C,D, the number of Gal3 puncta surrounded by the lysosome marker Lamp1 significantly increased with OGD treatment, in contrast to the diffuse distribution of puncta observed in the control group, indicating that the OGD treatment damaged the lysosomal membranes. As the data above strongly indicated the occurrence of lysosomal injury with OGD stress, we investigated whether OGD treatment simultaneously caused the release of cathepsins into the cytoplasm. We used digitonin to extract cytoplasm without lysosomes. We found a time-dependent increase in the activity of cytosolic Cat B, suggesting that it had leaked into the cytoplasm (Figure 2E). The results described above suggest that LMP occurred and might account for the cardiomyocyte loss in the group treated with OGD. Therefore, the specific cathepsin inhibitors pepstatin A (Cat D) and CA074 (Cat B) and Cat D siRNA (Figure 2J) were applied to combat the cell death caused by OGD stress. As expected, both the cathepsin inhibitors and Cat D siRNA increased cell survival under OGD stress, as detected by an increased CCK-8 level and a reduction in LDH release (Figures 2F–I).

**FIGURE 3:**
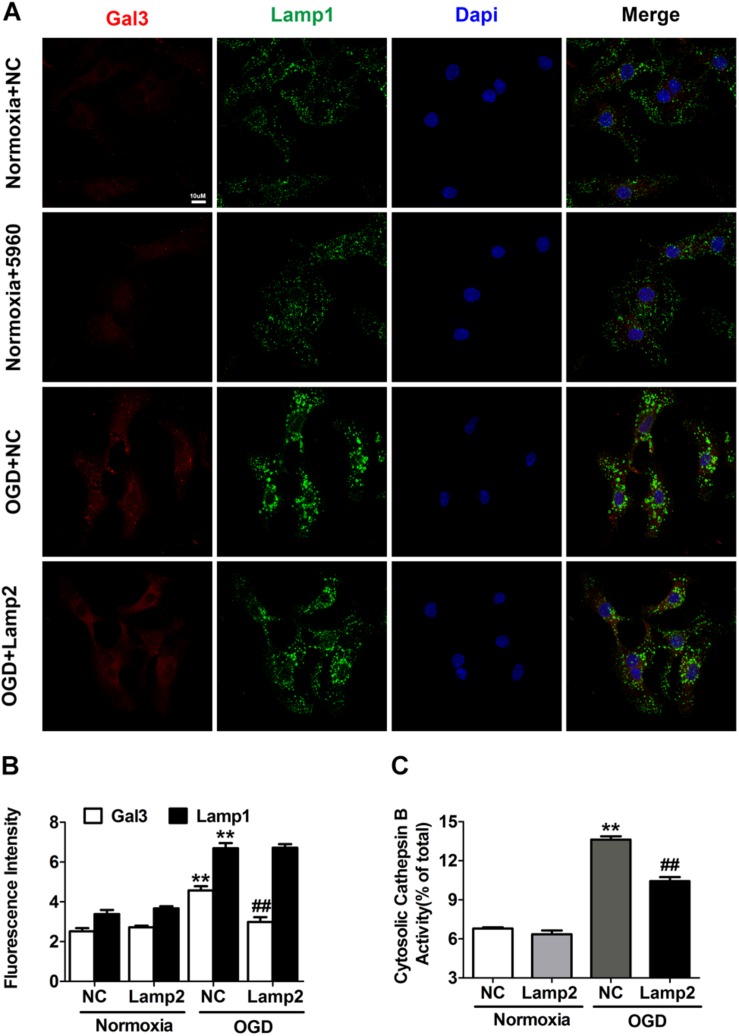
Exogenous overexpression of Lamp2 inhibits LMP in OGD-treated cardiomyocytes. **(A,B)** Representative confocal immunofluorescence images **(A)** and quantitative analysis of the Gal3 and Lamp1 level after OGD treatment for 9 h **(B)**. Scale bar, 10 μm. For the quantitative analysis, the data are presented as the means ± SEM (*n* = 3). **P < 0.01 versus the normoxia + NC group, ^##^*P* < 0.01 versus the OGD + NC group. **(C)** Detection of the activity of cytosolic cathepsin B without lysosomal fractions. Mean ± SEM. *n* = 3. **P < 0.01 versus the normoxia + NC group, ^##^*P* < 0.01 versus the OGD + NC group.

Next, we infected cardiomyocytes with adenovirus carrying full-length Lamp2 to determine its effect on LMP in response to OGD treatment. As shown in Figures 3A,B, Lamp2 overexpression notably reversed the increase in Gal3 puncta surrounded by Lamp1 that had been caused by OGD treatment. We further used Flag to detect the successfully transfected cardiomyocytes, and similarly, we discovered that cardiomyocytes with Flag expression had weaker Gal3 fluorescence intensity (Supplementary Figure S2), which indicated that Lamp2 ameliorated the injury to the lysosomal membranes. To further validate that restoration of Lamp2 inhibited LMP, we examined the activity of cytosolic Cat B. Similar to the findings using Gal3, exogenous expression of Lamp2 significantly prevented the increase in the activity of cytosolic Cat B with OGD treatment (Figure 3C). We also used siRNA to further downregulate the protein content of Lamp2 (Supplementary Figure S1A). In contrast, Lamp2 siRNA didn’t further exacerbate LMP with OGD stress but caused the injury of the lysosomal membranes in normal conditions (Supplementary Figures S1D–F). These data support the hypothesis that decrease of the protein level of Lamp2 renders the lysosomal membrane more vulnerable and its restoration inhibits the occurrence of LMP, which could explain the manner by which it protects against ischemic/hypoxic injury.

### Lamp2 Overexpression Reverses the Autophagic Flux Blockade Induced by OGD Stress

**FIGURE 4:**
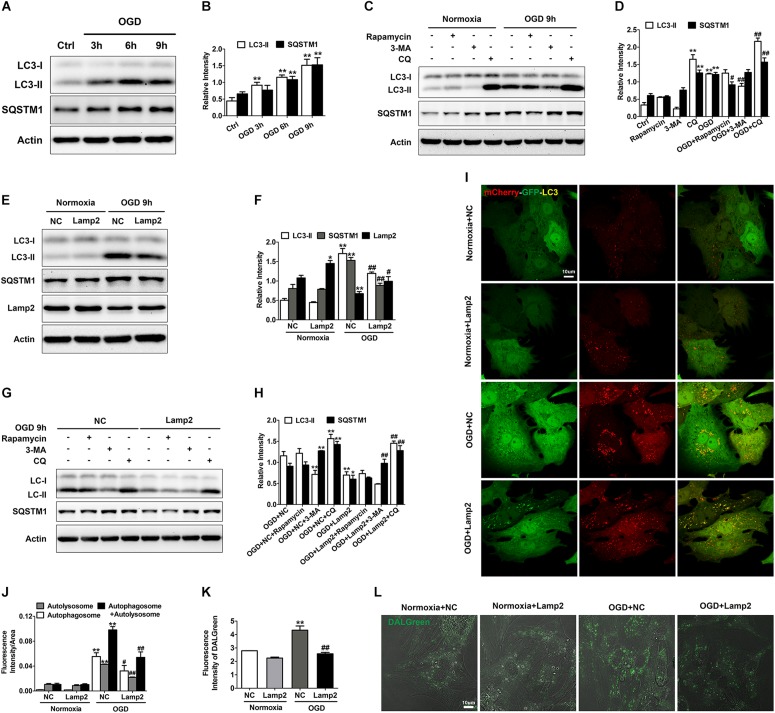
Lamp2 restoration rescues the autophagic flux blockade caused by OGD treatment. **(A,B)** Western blotting **(A)** and quantitative analysis **(B)** of LC3 and SQSTM1 in response to OGD treatment for 3, 6, and 9 h. Mean ± SEM (*n* = 3). **P < 0.01 versus the control group. **(C,D)** Western blotting **(C)** and quantitative analysis **(D)** of LC3 and SQSTM1 with rapamycin (200 nM), 3-MA (1 mM) and CQ (20 μM) treatment. Mean ± SEM (*n* = 3). **P < 0.01 versus the control group and ^#^*P* < 0.05, ^##^*P* < 0.01 versus the OGD group. **(E,F)** Western blotting of LC3, SQSTM1 and Lamp2 with exogenously infected full-length Lamp2 adenovirus **(E)** and results from the quantitative analysis **(F)**. Mean ± SEM (*n* = 3). *P < 0.05, **P < 0.01 versus the normoxia + NC group and ^#^*P* < 0.05, ^##^*P* < 0.01 versus the OGD + NC group. **(G,H)** Cardiomyocytes were treated with rapamycin, 3-MA or CQ with or without Lamp2 overexpression and Western blotting was performed to determine the levels of LC3 and SQSTM1 **(G)**. Quantitative analysis **(H)** of these results is presented as the mean ± SEM (*n* = 3). *P < 0.05, **P < 0.01 versus the OGD + NC group and ^##^*P* < 0.01 versus the OGD + Lamp2 group. **(I)** Representative confocal images of mCherry-GFP-LC3. Bar, 10 μm. **(F)** Quantitative analysis of **(J)** is presented as the mean ± SEM (*n* = 3). **P < 0.01 versus the normoxia + NC group and ^#^*P* < 0.05, ^##^*P* < 0.01 versus the OGD + NC group. **(L)** DALGreen fluorescent dye detection. Scale bar, 10 μm. **(K)** Quantitative analysis of **(L)**. Mean ± SEM (*n* = 3). **P < 0.01 versus the normoxia + NC group, ^##^*P* < 0.01 versus the OGD + NC group.

Enhanced autophagy may be a double-edged sword for the ischemic heart because it supplies energy to cells coupled by accumulated autophagosomes and autolysosomes. As shown in Figures 4A,B, autophagic markers (LC3-II and SQSTM1/p62) increased gradually after exposure to OGD. The abundance of autophagosomes and autolysosomes was assessed with mCherry-GFP-tagged LC3 adenovirus. Neonatal cardiomyocytes showed basal autophagy with a preponderance of autolysosomes, while OGD treatment markedly increased the abundance of both autophagosomes and autolysosomes (Figures 4I,J). The immunostaining of LC3 showed similar results (Supplementary Figure S3). Although the ischemic promotion of autophagy has been studied extensively in various cell types, the gradual accumulation of SQSTM1/p62 might reflect insufficient degradation of autophagy substrates. To clarify whether autophagic flux blockade accounted for the accumulated LC3-II and SQSTM1/p62, we used different drugs to further activate and inhibit autophagy respectively. As shown in Figures 4C,D, 3-methyladenine (3-MA) treatment partially reversed the increase of LC3-II caused by OGD treatment while rapamycin didn’t further increase its protein level. Although CQ treatment significantly increased LC3-II and SQSTM1/p62 in both normal and OGD conditions, the extent of their increases in the OGD group was much smaller than that of the normal group. These results suggest that the rate of autophagy induction significantly outpasses that of the autophagosome turnover, which causes the blockade of the autophagic flux and the accumulation of autophagosomes and autolysosomes.

Because Lamp2 is an important protein for the progression of autophagy, we aimed to determine whether its restoration in the cell could alleviate the autophagic flux blockade created by OGD stress. We found that the protein levels of LC3-II and SQSTM1/p62 were dramatically reduced and that the fluorescence intensity of LC3 was remarkably weakened when Lamp2 was overexpressed (Figures 4E,F, and Supplementary Figure S3). To further clarify whether this reduction was due to autophagy inhibition or accelerated autophagosomal and autolysosomal clearance, we applied different drugs to stimulate and inhibit autophagy with or without Lamp2 overexpression. As shown in Figures 4G,H, 3-MA further decreased the level of LC3-II in the Lamp2-overexpressing cells while rapamycin didn’t increase its protein content. CQ treatment caused significant increase in the protein levels of LC3-II and SQSTM1/p62 in OGD group with or without Lamp2 overexpression. These results implied that autophagy induction was not blocked with Lamp2 overexpression. Considering that Lamp2 is important for the fusion between autophagosomes and lysosomes, Lamp2 overexpression in the presence of OGD was expected to cause a reduction in autophagosomes and the accumulation of autolysosomes, as it has been reported that ischemia results in lysosomal dysfunction of the myocardium (Roy and Stanely Mainzen Prince, 2012). However, it was interesting to observe that the number of both autophagosomes and autolysosomes was remarkably decreased with Lamp2 overexpression in OGD group and Lamp2 knockdown in the control group caused accumulation of both autophagosomes and autolysosomes (Figures 4I,J and Supplementary Figures S1B,C). The reduction in autolysosomes with Lamp2 overexpression was further corroborated by the decrease in the fluorescence intensity of DALGreen (Figures 4K,L). Lamp2 knockdown in OGD group didn’t induce further accumulation of autophagic structures but caused the shift to more autophosomes and fewer autolysosomes (Supplementary Figures S1B,C). These data collectively reveal that, in addition to enhancing the fusion between autophagosomes and lysosomes, restoration of Lamp2 in OGD conditions also accelerates the clearance of autolysosomes.

### Lamp2 Improves the Trafficking Deficit of Cathepsins Caused by OGD Treatment

**FIGURE 5:**
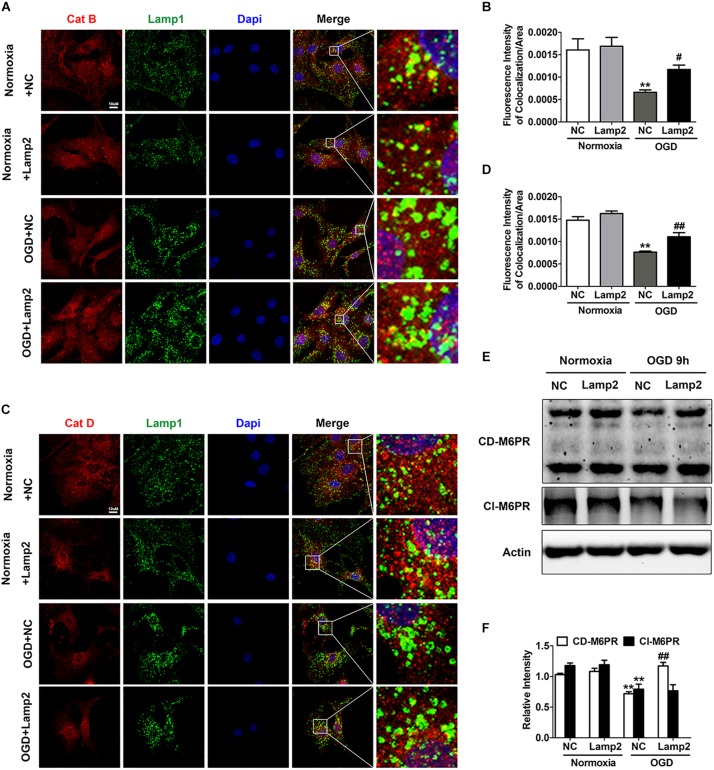
Overexpression of Lamp2 alleviates dysfunction of the cathepsin trafficking induced by OGD treatment. **(A,B)** Double staining of cathepsins and Lamp1 to determine the trafficking of cathepsin D **(A)** and cathepsin B **(B)**. Scale bar, 10 μm. **(C,D)** Quantitative analysis of **(A,B)**. Mean ± SEM (*n* = 3). **P < 0.01 versus the normoxia + NC group and ^#^*P* < 0.05, ^##^*P* < 0.01 versus the OGD + NC group. **(E,F)** Western blotting was performed to determine the protein levels of CD-M6PR and Cl-M6PR with Lamp2 overexpression **(E)**. Quantitative analysis **(F)** is presented as the mean ± SEM (*n* = 3). **P < 0.01 versus the normoxia + NC group, ^##^*P* < 0.01 versus the OGD + NC group.

It is relatively well documented that Lamp2 improves autophagic flux by promoting the fusion between autophagosomes and lysosomes (Hubert et al., 2016; Zhang et al., 2018). The accumulation of autolysosomes induced by OGD stress (Figure 4I and Supplementary Figure S1B) suggested that the degradation capacity of lysosomes overloaded with a robust increase in autophagic structures was deficient. Overexpression of Lamp2 further enhanced the fusion of autophagosomes and lysosomes but remarkably reduced the accumulation of autolysosomes, which strongly suggests that Lamp2 might play a role in enhancing the degradation capacity of lysosomes. Consequently, we investigated the subcellular localization of Cat B and Cat D and found that relatively more of these two cathepsins were distributed to Lamp1-positive endosomes and lysosomes in cells with Lamp2 overexpression (Figures 5A–D), indicating enhanced trafficking of these two cathepsins. Since cation-dependent mannose 6-phosphate receptor (CD-M6PR) and cation-independent mannose 6-phosphate receptor (Cl-M6PR) are two proteins known to be crucial for the trafficking of lysosomal hydrolases, we next examined the levels of these proteins. As shown in Figures 5E,F, the CD- M6PR protein level prominently increased with no significant change in the Cl-M6PR protein level in cells overexpressing Lamp2. The recovery of the CD-M6PR protein level might explain the improved trafficking of lysosomal cathepsins after Lamp2 restoration.

### Impaired Autophagic Flux Contributes to the Occurrence of LMP and Cell Death After OGD Treatment

**FIGURE 6:**
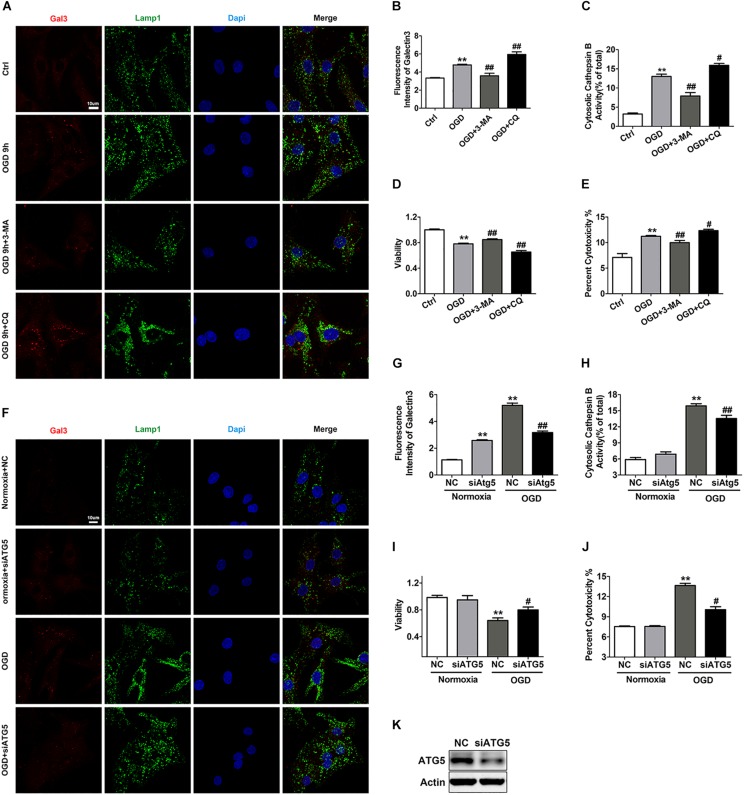
Autophagic flux blockade accounts for the OGD-induced LMP in cardiomyocytes. **(A,B)** Immunostaining of Gal3 after treatment with OGD with 3-MA (1 mM) or CQ (20 μM) **(A)**. Quantitative analysis **(B)**. Scale bar, 10 μm. Mean ± SEM (*n* = 3). **P < 0.01 versus the control group, ^##^*P* < 0.01 versus the OGD group. **(C)** The extent of LMP with 3-MA (1 mM) or CQ (20 μM) treatment was determined by the activity of cytosolic cathepsin B. Mean ± SEM (*n* = 3). **P < 0.01 versus the control group and ^#^*P* < 0.05, ^##^*P* < 0.01 versus the OGD group. **(D)** A CCK-8 assay was performed to evaluate cell viability. Mean ± SEM (*n* = 3). **P < 0.01 versus the control group, ^##^*P* < 0.01 versus the OGD group. **(E)** Cytotoxicity was determined by LDH leakage experiments. Mean ± SEM (*n* = 5). **P < 0.01 versus the control group, ^#^*P* < 0.05, ^##^*P* < 0.01 versus the OGD group. **(F,G)** Representative images of Gal3 with ATG5 siRNA **(F)**. Quantitative analysis **(G)**. Scale bar, 10 μm. Mean ± SEM (*n* = 3). **P < 0.01 versus the normoxia + NC group and ^##^*P* < 0.01 versus the OGD + NC group. **(H)** Detection of the activity of cytosolic cathepsin B after transfection with ATG5 siRNA. Mean ± SEM (*n* = 3). **P < 0.01 versus the normoxia + NC group and ^##^*P* < 0.01 versus the OGD + NC group. **(I)** Cell viability was detected by CCK-8 assay. Mean ± SEM (*n* = 4). **P < 0.01 versus the normoxia + NC group, ^#^*P* < 0.05 versus the OGD + NC group. **(J)** Cytotoxicity was assessed by LDH leakage experiments. Mean ± SEM (*n* = 3). **P < 0.01 versus the normoxia + NC group and ^##^*P* < 0.01 versus the OGD + NC group. **(K)** Western blotting was performed to detect the transfection efficiency of ATG5 siRNA.

Considering that autolysosomes that have accumulated due to autophagic flux blockade are larger in volume and more easily to be attacked, we set out to clarify whether impaired autophagic flux contributed to the LCD observed with OGD treatment. We applied 3-MA to alleviate the blockade of autophagic flux and discovered that 3-MA significantly reduced the number of Gal3 puncta and the activity of cytosolic Cat B (Figures 6A–C). Accordingly, 3-MA increased cell survival under OGD stress, as determined by the CCK-8 and LDH release experiments (Figures 6D,E). Conversely, in the CQ-treated OGD group, the fluorescence intensity of Gal3 puncta and cytosolic Cat B activity were greatly elevated (Figures 6A–C), indicating further reduction of cell viability (Figures 6D,E). ATG5 siRNA was also used to specifically inhibit autophagy and decrease the accumulation of autophagic structures (Figure 6K). As expected, ATG5 siRNA partially inhibited the occurrence of LMP with OGD treatment and alleviated cardiomyocyte injury (Figures 6F–J). These data suggests that autophagic flux impairment contributes to the occurrence of LMP and the subsequent LCD in response to OGD. LMP could in turn exacerbate lysosomal dysfunction, resulting in an even more severe autophagic flux blockade and creating a vicious cycle.

## Discussion

LMP has been reported to be involved in the cell death control in various physiological and pathological conditions (Windelborn and Lipton, 2008; Gomez-Sintes et al., 2016; Jiang et al., 2016; Gaidt et al., 2017; Mai et al., 2017; Lin and Hardie, 2018), but its role in myocardial ischemic injury remains uncertain. In the present study, we used different methods to demonstrate the occurrence of LMP in ischemic cardiomyocytes *in vitro*. With the application of inhibitors and siRNA of cathepsins, we demonstrated that LMP was involved in the regulation of cardiomyocyte death induced by OGD treatment. We further uncovered the underlying mechanisms of LMP and cardiomyocyte injury caused by OGD treatment.

Previous studies have reported that Lamp2 confers stress resistance against various conditions, including acidosis, oxidative stress and neurodegenerative disorders (Saha, 2012; Damaghi et al., 2015; Qin et al., 2017; Issa et al., 2018) and is recognized as a vital protector against LMP with unclear underlying mechanisms (Geronimo-Olvera et al., 2017; Wiedmer et al., 2017). In our experimental system, OGD treatment caused the decline of Lamp2 and restoration of Lamp2 partially reduced LMP and cell death of cardiomyocytes. Lamp2 siRNA in normal conditions caused the injury of lysosomal membranes and the decline of cardiomyocytes viability. These results indicated that Lamp2 is involved in the regulation of LMP and cell death and confers cardiomyocytes resistance against OGD treatment. Cardiac ischemia is a very complicated pathological condition and many factors are involved in the regulation of cardiomyocyte injury, including the reactive oxygen species (ROS) overload, mitochondria dysfunction and so on (Ganitkevich et al., 2006; Pasdois et al., 2012; Jian et al., 2016; Pei et al., 2017). Lamp2 could be one of the protective factors although its effect seemed relatively modest. However, further downregulation of Lamp2 with siRNA in OGD condition didn’t exacerbate LMP or cell death. Considering that all patients of Danon disease including female patients with decreased Lamp2 expression due to a heterozygous null mutation and male patients with completely absent Lamp2 expression develop severe cardiomyopathy (Endo et al., 2015; Sugie et al., 2018; Cenacchi et al., 2019), we can deduce that Lamp2 is especially important for the proper function of the heart and that Lamp2 couldn’t function properly and act as a protectant when its protein content decreased to a certain degree. Consequently, it is possible that further reduction with siRNA in OGD group couldn’t cause more injury. In addition, feedback mechanisms are common among organisms with various injuries and further downregulation of Lamp2 might also promote the expression of other protective proteins, which compensated for its decrease.

Lamp2 is also known to promote autophagic flux and samples from patients of Danon disease present accumulated LC3 positive autophagosomes and undigested lipofuscin, indicating defective autophagic flux (Tanaka et al., 2000; Endo et al., 2015; Giuliano et al., 2015). An *in vitro* study of Lamp2-deficient mouse embryonic fibroblasts discovered that Lamp2 deficiency leads to the depletion of syntaxin-17 from autophagosomes and consequently defective fusion between lysosomes and autophagosomes (Hubert et al., 2016). Cardiac ischemia/reperfusion injury causes a decrease in Lamp2 and a consequent blockade of autophagic flux (Ma et al., 2012). In our study, we also found a blockade of autophagic flux reflected by accumulated autophagosomes and autolysosomes in OGD conditions or in normal conditions with Lamp2 siRNA. Exogenous expression of Lamp2 restored the impaired autophagic flux. However, further downregulation of Lamp2 in OGD group caused the shift to more autophagosomes and fewer autolysosomes without significant changes of the overall autophagic structures, indicating that further reduction of Lamp2 caused fully inhibition of the fusion between autophagosomes and lysosomes. Considering that lysosomes with larger volume are more vulnerable to various risk factors (Ono et al., 2003), this result might also explain why Lamp2 siRNA in OGD group didn’t further exacerbate LMP and cell death as the reduction of autolysosomes decreased the volume and vulnerability of lysosomes, which compensated for other damages caused by the reduction of Lamp2. Cardiomyocytes overexpressing Lamp2 presented a remarkable reduction in autolysosomes with accelerated fusion between autophagosomes and lysosomes, which suggested its role in enhancing the hydrolytic function of lysosomes in response to OGD stress. The results that the protein level of CD-M6PR was restored and that the trafficking of Cat B and Cat D increased with the exogenous expression of Lamp2 confirmed our assumption. This finding was also consistent with the study that Lamp2-deficient hepatocytes present accumulation in both early- and late-stage autophagic vacuoles accompanied by a decrease in the activity of lysosomal enzymes and an abnormal reduction of mannose-6-phosphate receptors (Eskelinen et al., 2002). However, the manner by which Lamp2 correlates with CD-M6PR remains to be elucidated. Previous studies have found that chronic acidosis–induced Lamp2 overexpression in cancer cells confers plasma membrane resistance against acidosis-induced proteolysis (Damaghi et al., 2015). It is possible that Lamp2 overexpression slows the degradation rate of CD-M6PR by lysosomes. To avoid being digested, CD-M6PR needs to detach from endosomes and be recycled back to the Golgi complex by its adaptors (Diaz and Pfeffer, 1998; Nair et al., 2003), and Lamp2 might accelerate this process by stabilizing the CD-M6PR adaptors.

The role of autophagy in ischemia and ischemia/reperfusion injury is still debated. Excessive inhibition of Beclin1 markedly increases cardiomyocyte death in response to hypoxia/reoxygenation injury (Issa et al., 2018). However, it has also been reported that different mechanisms to inhibit autophagy also protect against myocardial ischemia/reperfusion injury (Wu et al., 2018; Yu et al., 2018). It has been found that application of 3-MA to inhibit autophagy alleviates astrocyte injury by inhibiting LMP (Zhou et al., 2017). In the present study, we also observed that 3-MA treatment and ATG5 siRNA improved cardiomyocyte resistance against OGD stress and inhibited LMP, whereas application of CQ to impede autophagic flux further exacerbated LMP. These results suggested that the autophagic flux blockade induced by OGD stress prompted LMP and the consequent LCD in cardiomyocytes. We also observed that Lamp2 siRNA in control group caused the increase in both autophagosomes and autolysosomes and the induction of LMP, while Lamp2 siRNA in OGD group didn’t exacerbated LMP with no further accumulation of autolysosomes. It is possible that various methods to reduce the quantity of the undigested autolysosomes lowers the volume of lysosomes and their vulnerability to be destructed.

## Conclusion

**FIGURE 7:**
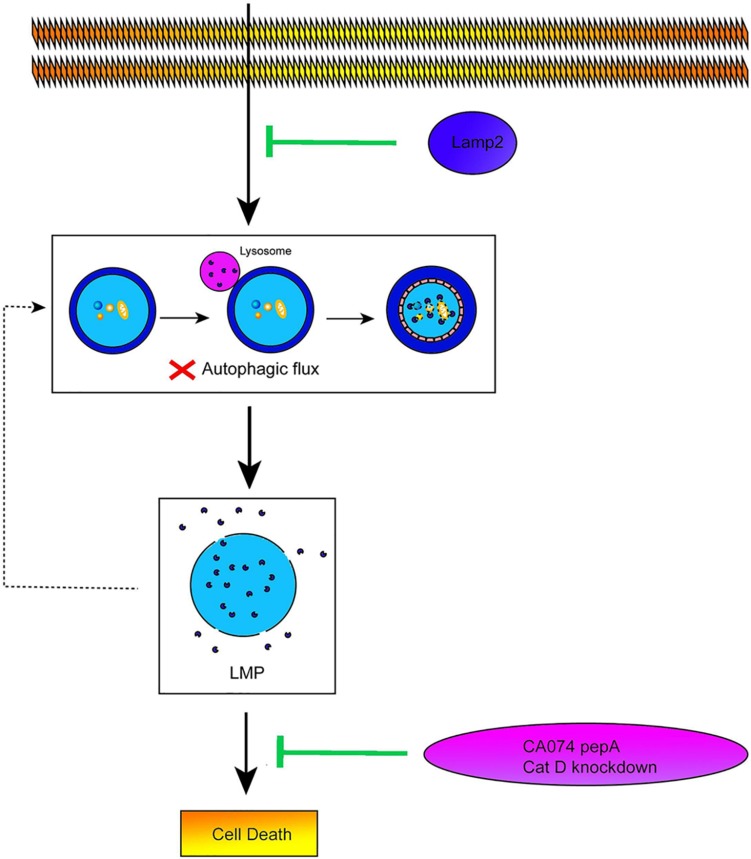
Lamp2 protects cardiomyocytes from OGD treatment. Schematic diagram on the proposed pathway that Lamp2 improved the impaired autophagic flux and thus alleviates LMP and cell death of cardiomyocytes caused by OGD treatment.

The data obtained from our study revealed that restoration of the Lamp2 protein alleviated the autophagic flux blockade induced by OGD stress by improving Cat B and Cat D trafficking, which inhibited LMP and conferred cardiomyocyte resistance against OGD stress (Figure 7). Although overexpression of Lamp2 partially alleviated LMP and cell death in the *in vitro* ischemic model, it still remains to be determined its contribution to the *in vivo* mechanism involving the myocardial ischemic injury. Considering that Lamp2 is indispensable for the proper function of myocardium and protects against various peroxidation injuries, it may be a potential therapeutic target against myocardial ischemic injury.

## Data Availability Statement

All data sets generated for this study are included in the article/Supplementary Material.

## Author Contributions

LC carried out the experimental work, collected the data, and drafted the manuscript. L-PZ and J-YY participated in the design and coordination of the experimental work. LY and YH participated in the experimental work and the analysis of the data. X-PJ participated in the study design and the interpretation of the data. QZ and J-ZJ participated in the coordination of the experimental work. D-XZ and YH carried out the study design, the analysis and interpretation of the data, and the modification of the manuscript.

## Conflict of Interest

The authors declare that the research was conducted in the absence of any commercial or financial relationships that could be construed as a potential conflict of interest.

## Abbreviations

3-MA: 3-methyladenine
Cat B: cathepsin B
Cat D: cathepsin D
CCK8: cell counting kit-8
CD-M6PR: cation-dependent mannose 6-phosphate receptor
Cl-M6PR: cation-independent mannose 6-phosphate receptor
CQ: chloroquine
Gal3: galectin-3
Lamp1: lysosomal-associated membrane protein
Lamp2: lysosomal membrane-associated protein 2
LAMPs: lysosomal-associated membrane proteins
LCD: lysosomal cell death
LDH: lactate dehydrogenase
LMP: lysosomal membrane permeabilization
OGD, oxygen: glucose and serum deprivation
ROS: reactive oxygen species
