# Supplementary material for: The Lysosomal Membrane Protein Lamp2 Alleviates Lysosomal Cell Death by Promoting Autophagic Flux in Ischemic Cardiomyocytes
Source: Front Cell Dev Biol. 2020 Feb 7;8:31. doi: 10.3389/fcell.2020.00031 (PMC7019187; doi:10.3389/fcell.2020.00031)
Supplement: Supplementary file 1 [file Data_Sheet_1.pdf]

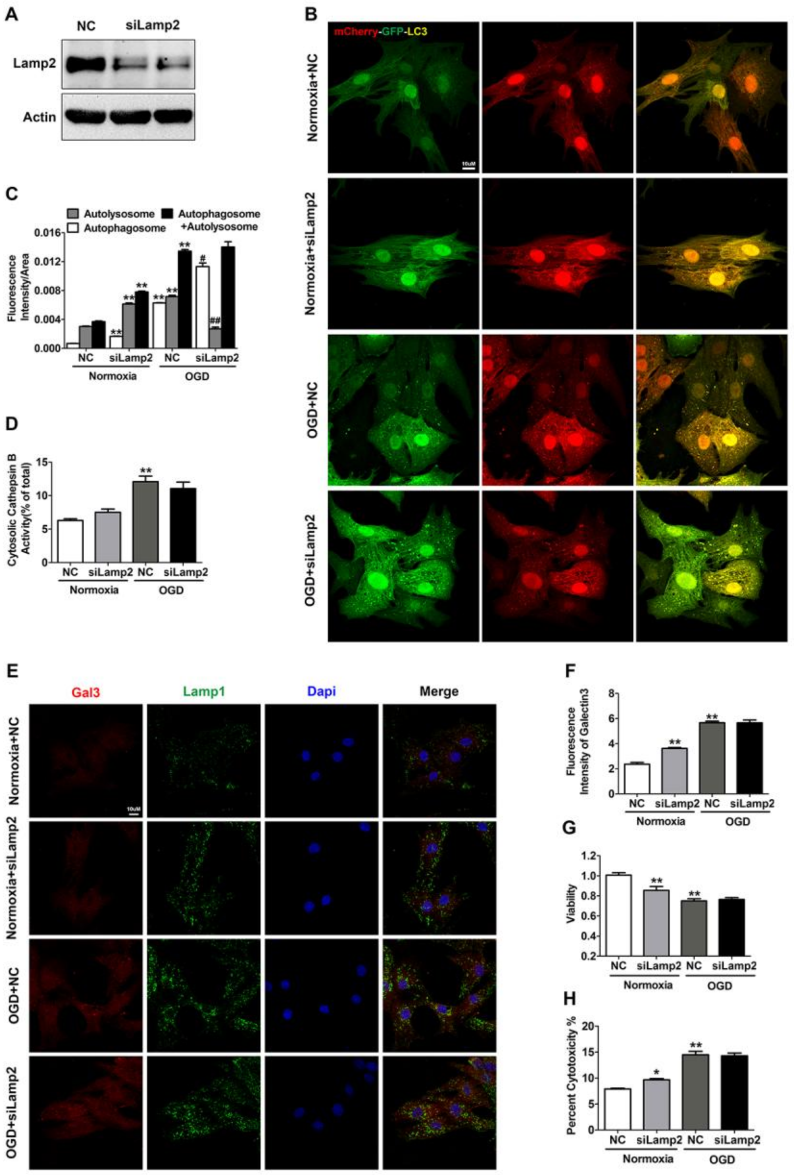

Supplementary Figure 1

A) Western blotting was performed to detect the transfection efficiency of Lamp2 siRNA. *B*) Representative confocal images of mCherry-GFP-LC3. Bar, 10 μm. *C*) Quantitative analysis of (*B)* is presented as the mean ±SEM (n=3). **P<0.01 versus the normoxia+NC group and #P<0.05, ##P<0.01 versus the OGD+NC group. *D*) Detection of the activity of cytosolic cathepsin B after transfection with Lamp2 siRNA. Mean ±SEM (n=3). **P<0.01 versus the normoxia+NC group. *E* and *F*) Representative images of Gal3 with Lamp2 siRNA (*E*). Quantitative analysis (*F*). Scale bar, 10 μm. Mean ± SEM (n=3). **P<0.01 versus the normoxia+NC group. *G*) Cell viability was detected by CCK-8 assay. Mean ± SEM (n=3). **P<0.01 versus the normoxia+NC group. *H*) Cytotoxicity was assessed by LDH leakage experiments. Mean ± SEM (n=3). *P<0.05, **P<0.01 versus the normoxia+NC group.

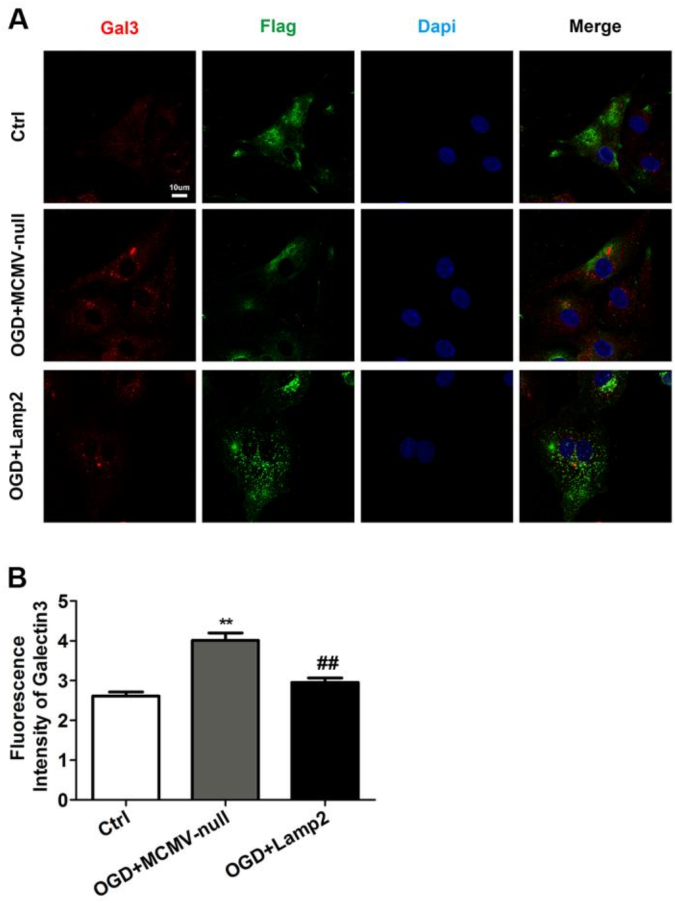

Supplementary Figure 2

A) Representative confocal images of Gal3. Flag was used to detect cells that were successfully transfected. Scale bar, 10um. *B*) Quantitative analysis of (*A*) and the data were presented as Means ± SEM (n=22). **P<0.01 versus the control group, $^{##}$P<0.01 versus the OGD+MCMV-null group.

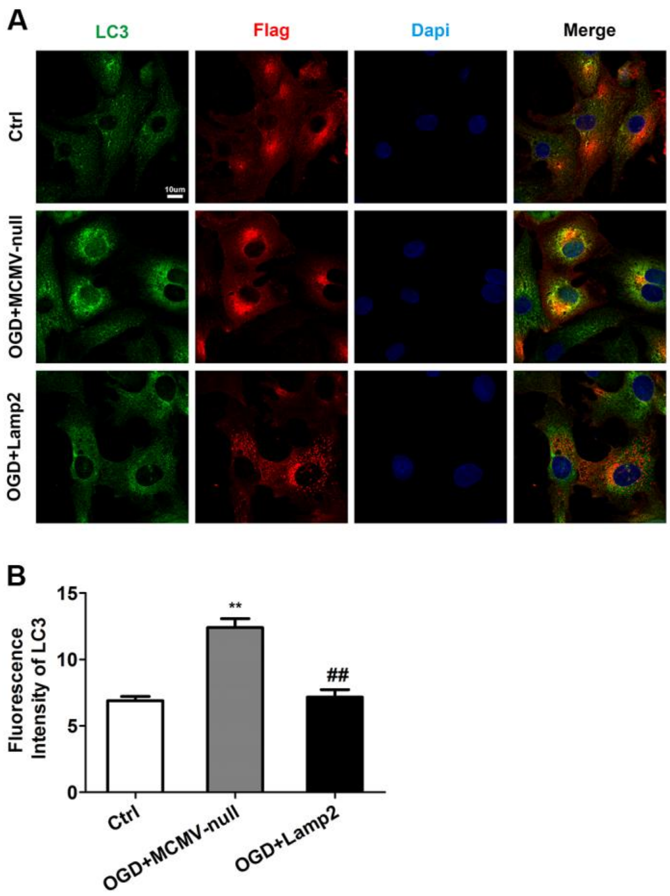

Supplementary Figure 3

A) Immunostaining of LC3 was performed to detect autophagy and Flag positive represented cells that were successfully transfected. Bar, 10um. *B*) Quantitative analysis of (*A*) and the data were presented as Means ± SEM (n=21). **P<0.01 versus the control group, ##P<0.01 versus the OGD+MCMV-null group.
